# Editorial Note

**Published:** 1912-09

**Authors:** 


### Editorial Note.

**The Whole-Time Tuberculosis Officer**

It is much to be regretted that the attention of the profession has been so concentrated upon the medical benefit sections of the Insurance scheme that its Sanatorium benefit clauses have been, comparatively speaking, ignored. Had it been subjected to more criticism, we feel sure that the whole-time officer would have been statutorily limited to his sphere of real usefulness, that of administration. Even the short and restricted criticism which has been brought to bear upon the matter has made a good deal of difference in this direction. At first the whole-time officer was destined to undertake wide responsibilities in the diagnosis and treatment of tuberculosis; now, however, we find from the latest order of the Local Government Board, that he is not to carry out domiciliary treatment at all except through a rather absurdly stereotyped series of printed forms, which the general practitioner treating the patient is to fill up and forward periodically to the whole-time officer. We are convinced that sooner or later this evolution of the whole-time officer will be carried to its logical conclusion, limitation to administrative work by the pressure of natural forces.

Specialism is an unfortunate necessity in certain territories of medicine, but tuberculosis, with its incidence on all sorts and conditions of organs and tissues, cannot be mapped out like one of these. The best clinical unit for the struggle with tuberculosis in all its forms is an efficient general practitioner, with a group of consultants within reach; and if special training in the diagnosis of pulmonary tuberculosis were insisted on in the education of every medical student, it would repeat on the wholesale scale all the good which is obtained in a sporadic form from the now popular pilgrimage to Edinburgh.

But the " tuberculosis specialist " is likely to prove injurious as well as unnecessary. He is to be paid to find tuberculosis, and he will have to justify his existence by presenting turgid masses of statistics to his committee. Small wonder if cases which would be " doubtful " and " negative " to a less prejudiced observer are written down by him as " positive," regardless of the miserable anxiety to patient and friends which is the inevitable result. It is, therefore, clearly in the public interest that no notification of tuberculosis be made by the whole-time officer which has not been ratified by some other medical man. The real onus of early diagnosis must always fall on the general practitioner. He alone knows the make-up and the surroundings of the patient ; it is to him that the tuberculous person will turn at first, feeling ill but not knowing why ; and an army of well-taught family doctors can carry on a far wider campaign of examination of contacts than can be attempted by a group of officials.

The position of the whole-time officer, then, is this. Direct responsibility for treatment has already been taken away from him by the Local Government Board, while he is disqualified for early diagnosis by the immensity and the intimacy of the task. What excuse remains for his existence ? We think he will have a real administrative value ; it will be an advantage to incorporate in one officer a knowledge both of the demand for institutional and other treatment, and of the supply available at any given moment. No doubt also the medical officer of health, whose assistant he will be, will find uses for him. It is, therefore, to an administrative and not to a clinical career that the aspiring tuberculosis officer should look forward, for the clinical path which now appears to furnish so promising an opening is in reality a blind alley. It should never be forgotten that the Departmental Committee, whose interim report forms the basis of the Government scheme, was practically composed of medical officers of health, with one or two consumption specialists and no general practitioner, and the product of their deliberations is, not unnaturally, an assistant to the medical officer of health. This is the proper *métier* of the whole-time tuberculosis officer, and he should be limited to it. Whether the public will enjoy paying salaries to assistant medical officers of health is doubtful, but at any rate it is not our affair.

### Notes on Preparations for the Sick.

